# Compound heterozygous *POMT1* mutations in a Chinese family with autosomal recessive muscular dystrophy‐dystroglycanopathy C1

**DOI:** 10.1111/jcmm.13068

**Published:** 2017-02-03

**Authors:** Pengzhi Hu, Song Wu, Lamei Yuan, Qiongfen Lin, Wen Zheng, Hong Xia, Hongbo Xu, Liping Guan, Hao Deng

**Affiliations:** ^1^ Center for Experimental Medicine and Department of NeurologyThe Third Xiangya HospitalCentral South UniversityChangshaChina; ^2^ Department of OrthopedicsThe Third Xiangya HospitalCentral South UniversityChangshaChina; ^3^ BGI‐ShenzhenShenzhenChina

**Keywords:** muscular dystrophy‐dystroglycanopathy, exome sequencing, *POMT1* gene, mutation, genetic counselling

## Abstract

Muscular dystrophy‐dystroglycanopathy (MDDG) is a genetically and clinically heterogeneous group of muscular disorders, characterized by congenital muscular dystrophy or later‐onset limb‐girdle muscular dystrophy accompanied by brain and ocular abnormalities, resulting from aberrant alpha‐dystroglycan glycosylation. Exome sequencing and Sanger sequencing were performed on a six‐generation consanguineous Han Chinese family, members of which had autosomal recessive MDDG. Compound heterozygous mutations, c.1338+1G>A (p.H415Kfs*3) and c.1457G>C (p.W486S, rs746849558), in the protein O‐mannosyltransferase 1 gene (*POMT1*), were identified as the genetic cause. Patients that exhibited milder MDDG manifested as later‐onset progressive proximal pelvic, shoulder girdle and limb muscle weakness, joint contractures, mental retardation and elevated creatine kinase, without structural brain or ocular abnormalities, were further genetically diagnosed as MDDGC1. The *POMT1* gene splice‐site mutation (c.1338+1G>A) which leads to exon 13 skipping and results in a truncated protein may contribute to a severe phenotype, while the allelic missense mutation (p.W486S) may reduce MDDG severity. These findings may expand phenotype and mutation spectrum of the *POMT1* gene. Clinical diagnosis supplemented with molecular screening may result in more accurate diagnoses of, prognoses for, and improved genetic counselling for this disease.

## Introduction

Muscular dystrophy‐dystroglycanopathy (MDDG), a common group of muscular dystrophies, is clinically characterized by congenital muscular dystrophy (CMD) or later‐onset limb‐girdle muscular dystrophy (LGMD). It may occur with or without brain and ocular abnormalities 1, 2. Clinical signs include progressive muscle weakness with variable onset ages and phenotypic severity, hypotonia and markedly elevated serum creatine kinase (CK) levels. It can be accompanied by structural brain anomalies including agyria, hydrocephalus and cerebellar hypoplasia, or ocular defects such as cataracts, microphthalmia and buphthalmos. Mental retardation (MR) may be present 1, 3, 4, 5. Classical pathological features are dystrophic findings on muscle biopsy, and hypoglycosylated alpha‐dystroglycan (α‐DG) in skeletal muscle with glycosylated epitopes specific antibodies on the α‐DG 1, 2, 3, 6. The incidence and prevalence of CMD are unknown with a prevalence of 0.563/100,000 reported in Italy, and the estimated prevalence of rare LGMD is 0.07‐0.43/100,000, varying in different ethnicities 3, 4, 7. In 2010, three MDDG phenotypic groups, listed in alphabetical order, were proposed by Online Mendelian Inheritance in Man (OMIM), nominated as MDDG type A (Walker–Warburg syndrome and Walker–Warburg syndrome‐like, muscle–eye–brain disease and Fukuyama CMD‐like), MDDG type B (CMD with cerebellar involvement, CMD with MR and CMD with no MR) and MDDG type C (LGMD with MR and LGMD with no MR) 1, 2, 8. Genetic defects causing aberrant post‐translational modification of α‐DG may be involved in the pathogenic mechanism 5, 8. This tripartite subdivision of MDDG is further indicated numerically according to disease‐associated genes 2, 8. To date, at least 12 genes, implicated in proper α‐DG glycosylation, and five genes, indirectly relating to dystroglycanopathy, are reported to be associated with MDDG 4, 9. Mutations in the protein O‐mannosyltransferase 1 gene (*POMT1*, OMIM 607423) are related to autosomal recessive muscular dystrophies, which include three subtypes, designated as MDDGA1 (OMIM 236670), MDDGB1 (OMIM 613155) and MDDGC1 (also known as LGMD2K, OMIM 609308) 2, 5. This study was aimed to identify the genetic defects responsible for MDDG in a Han Chinese family. Compound heterozygous mutations in the *POMT1* gene, including a previously described splice‐site mutation (c.1338+1G>A) involving in splicing of precursor mRNA, and a missense mutation (c.1457G>C, p.W486S) in a highly conserved region, were detected to be the possible genetic aetiology of MDDGC1 in this family.

## Materials and methods

### Participants and clinical description

Members of a six‐generation consanguineous Han Chinese family with putative muscular dystrophy were enrolled for genetic analysis at the Third Xiangya Hospital, Central South University, Changsha, China. General physical examinations and thorough neurological examinations of all the available family members were performed. Other conditions, including progressive proximal pelvic, shoulder girdle and limb muscle weakness after having acquired ambulation, joint contractures, and moderate or mild MR, revealed a more refined diagnosis 2, 4, 5. Clinical data, including age, gender, onset age, muscular dystrophy symptoms, and intellectual evaluation, and auxiliary examinations including CK levels, ophthalmologic examination, electromyography, brain imaging and muscle imaging were also collected (Table 1). Systemic metabolic and other acquired conditions were excluded. However, muscular biopsy in the patients was unavailable as they refused. One hundred unrelated ethnically matched healthy volunteers who did not have either similar symptoms of or a positive history for this condition took part in this study. After written informed consents were signed, peripheral blood used for genetic testing was sampled from all the available family members and the controls. The Institutional Review Board of the Third Xiangya Hospital approved the study, which was conducted in compliance with the Declaration of Helsinki principles. A standard phenol–chloroform extracting procedure was used to isolate genomic DNA (gDNA) from peripheral blood 10.

**Table 1:** Clinical characteristics and auxiliary examinations of family members with the *POMT1* gene mutation

Subject	IV:4	V:2	V:5	V:6	VI:2	VI:3
Gender	Female	Female	Female	Male	Female	Female
Age (years)	81	46	43	44	17	13
Genotype	Heterozygote: c.1457G>C	Heterozygote: c.1338+1G>A	Heterozygote: c.1338+1G>A	Heterozygote: c.1457G>C	Compound heterozygotes: c.1338+1G>A and c.1457G>C	Compound heterozygotes: c.1338+1G>A and c.1457G>C
Onset age (years)	No	No	No	No	2	3
Symptoms at onset	No	No	No	No	Difficulty in running and walking, and frequent fallings after having acquired ambulation	Difficulty in running and walking, and frequent fallings after having acquired ambulation
Muscle atrophies (MRI)	NA	No	No	No	Lower and upper limbs	No
Muscle hypertrophy	No	No	No	No	Left lower limb	No
Muscle weakness	No	No	No	No	Yes	Yes
Joint contractures	No	No	No	No	Yes (elbow and ankle joints)	Yes (ankle joints)
Mental retardation	No	No	No	No	Yes (moderate)	Yes (mild)
Serum creatine kinase level	Normal	Normal	Normal	Normal	Elevated	Elevated
Electromyography	NA	NA	NA	NA	Myopathy	Myopathy
Ocular examination	Normal	Normal	Normal	Normal	Normal	Normal
Brain structure (MRI)	NA	Normal	Normal	Normal	Fifth and sixth cerebral ventricles	Normal

*POMT1*: the protein O‐mannosyltransferase 1 gene; NA: not available; MRI: magnetic resonance imaging.

### Exome library construction, reads alignments and variant analysis

Exome sequencing was conducted in the proband of this family (VI:2, Fig. 1A) on the Illumina HiSeq 2000 platform at BGI‐Shenzhen (Shenzhen, China), as previously described 11, 12. Single nucleotide polymorphisms (SNPs) and small insertions–deletions (indels) in coding sequences or splice sites were identified 10, 11, 12. Sorting Intolerant from Tolerant (http://sift.jcvi.org/) algorithm was used to test possible pathogenic function of non‐synonymous SNPs 13. Common and other non‐pathogenic candidate variants were sifted out using several public databases: database of SNPs (dbSNP version 137, http://www.ncbi.nlm.nih.gov/projects/SNP/snp_summary.cgi), 1000 genomes project (1000 genomes release phase 3, http://www.1000genomes.org/), HapMap project (2010‐08_phase II + III, http://hapmap.ncbi.nlm.nih.gov/), Exome Variant Server (EVS, http://evs.gs.washington.edu/EVS/) and in‐house exome BGI database. Only variants, which include non‐synonymous SNPs in exonic regions, coding indels or canonical splice‐site changes, can be regarded as pathogenic candidates 14.

**Figure 1:**
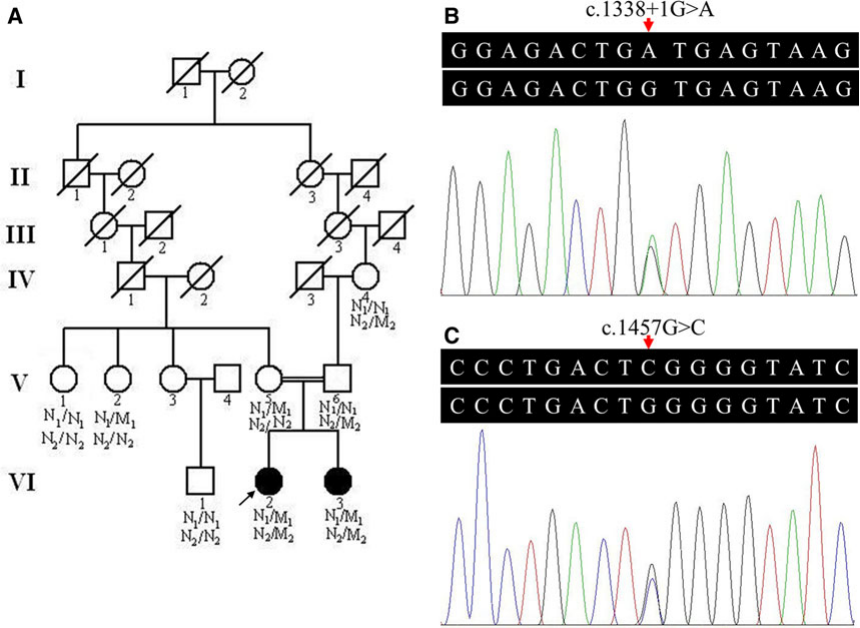
Pedigree and compound heterozygous mutations of a family with MDDGC1. (**A**) Pedigree of the MDDGC1 family. N_1_, N_2_: normal; M_1_: *POMT1* c.1338+1G>A mutation; M_2_: *POMT1* c.1457G>C mutation. The arrow indicates the proband. (**B**) and (**C**) Chromatograms (genomic DNA) of heterozygous *POMT1* c.1338+1G>A and c.1457G>C mutations in the affected proband (VI:2). MDDGC1: muscular dystrophy‐dystroglycanopathy C1; *POMT1*: the protein O‐mannosyltransferase 1 gene.

Locus‐specific polymerase chain reaction (PCR) amplification primers were designed for potential pathogenic variants, and Sanger sequencing was performed on the ABI3500 genetic analyzer (Applied Biosystems, Foster City, CA, USA) 15. Pathogenicity prediction of non‐synonymous substitutions was evaluated by other two programs: Polymorphism Phenotyping version 2 (http://genetics.bwh.harvard.edu/pph2/) and MutationTaster (http://www.mutationtaster.org/) 16, 17. The function of a potential splice‐site variant on splicing was predicted (http://www.fruitfly.org/seq_tools/splice.html) 18. Two pairs of primer sequences (NCBI reference sequence: NM_007171.3) are as follows: 5′‐GCAACCTTTTCCTGCCTGAA‐3′ and 5′‐GTGTTCTGTTAGGAAGTGCTCT‐3′, 5′‐GTTCCCCTTCCAACCCAAGT‐3′ and 5′‐TCAGTTCCCTTCCCACCAAA‐3′.

Total RNA was isolated from the lymphocytes of family members carrying variants to further determine whether the variants affect messenger RNA (mRNA). Complementary DNA (cDNA) was synthesized *via* reverse transcription PCR. PCR amplification was conducted using paired primers as follows: 5′‐CACGGGGACATGGTGCAG‐3′ and 5′‐AAGACAGCGGAAGTGTTCAC‐3′, 5′‐CTCTCAGAGGTCCGCTTTGT‐3′ and 5′‐TCGCCATGAAGCTGAGGTT‐3′ 15, 19.

## Results

**Figure 2:**
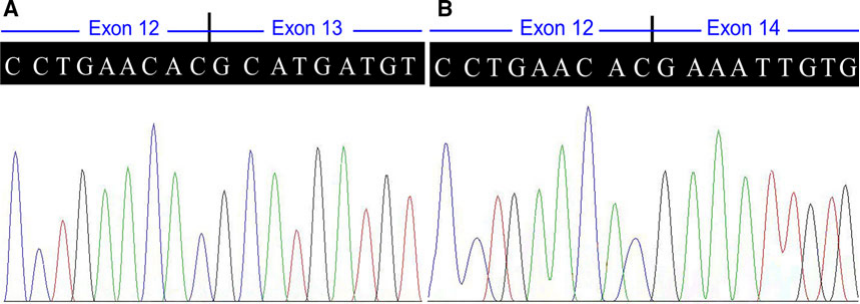
Sequence analysis of normal (**A**) and abnormal (**B**) splicing in the *POMT1* gene (complementary DNA) identified in the proband (VI:2). *POMT1*: the protein O‐mannosyltransferase 1 gene.

Exome sequencing in the proband produced about 69.42 million reads with a read length of 90 bp. There were 56.23 million reads aligned to the human genome; 2914.76 Mb were mapped to the target region with a mean coverage of 66.02×. There were 93,994 SNPs, including 11,153 non‐synonymous SNPs in the coding sequence and 2490 in the splice sites, detected. There were 7154 indels, including 412 in the coding sequence and 420 in the splice sites, identified. A prioritized filtration strategy of the variants was carried out following a scheme performed in previous studies 11, 12. Given the disorder's rarity, common variants identified in dbSNP137, 1000 genomes project, HapMap or EVS with a minor allele frequency higher than 0.50% were excluded. The remaining variants were further sifted out using in‐house BGI exome database with 2375 Chinese descent controls lacking similar symptoms. No homozygous variant was found, and only compound heterozygous variants (c.1338+1G>A and c.1457G>C) in the *POMT1* gene were identified in the proband, which were confirmed in both affected siblings (VI:2 and VI:3). Either no mutation or a heterozygous mutation (c.1338+1G>A or c.1457G>C) was observed in the unaffected family members (IV:4, V:1, V:2, V:5, V:6 and VI:1) and the 100 normal controls after further validation of Sanger sequencing (Fig. 1). The reverse transcription PCR and Sanger sequencing assay confirmed the online splice‐site prediction analysis that the splice‐site mutation, a G>A transition at position +1 of the 5′ splice donor site of intron 13, would lead to the loss of 5′ splice site. This mutation impairs *POMT1* precursor mRNA from correctly splicing and results in exon 13 being deleted (Fig. 2). A shift in the reading frame and a premature translation termination (p.H415Kfs*3) then result. *In silico* analysis predicted that the c.1457G>C (p.W486S) missense variant would be deleterious. Multiple sequence alignment revealed high conservation of tryptophan at position 486 (p.W486) in the homologous human protein POMT2 and in the POMT1 orthologs (http://blast.ncbi.nlm.nih.gov/Blast.cgi) 20. These findings support the possible pathogenicity of variants in this family.

## Discussion

Defective α‐DG glycosylation is the main factor responsible for CMD and LGMD, collectively termed MDDG, which is a genetically heterogeneous group of muscular dystrophies with a wide range of clinical severity 1, 2, 5. The variable phenotypic severity may be related to the type and location of the mutations, while there is no clear genotype–phenotype correlation 9, 21, 22. MDDGC1 is a mild subtype of autosomal recessive condition caused by the *POMT1* gene mutations 1, 2. It is characterized by proximal muscular weakness after having acquired ambulation, first involving the voluntary muscles of the hip, shoulder and limbs, with variable MR or mild brain anomalies 2, 7, 23.

In this research, compound heterozygous mutations, including a splice‐site mutation c.1338+1G>A (p.H415Kfs*3) and a missense mutation c.1457G>C (p.W486S) in the *POMT1* gene, were detected in members of an intermarriage Han Chinese family with MDDGC1 and cosegregated with the disease status. Generally, genetic disorders in consanguineous families are attributed to homozygous mutations. The compound heterozygous mutations identified in this family indicated that the causative factor was not associated with consanguineous marriage 10, 24. The mutations were absent in the 2475 ethnically matched unrelated controls who did not have similar symptoms, including 2375 controls obtained from the BGI exome sequencing and 100 normal controls in this study. *In silico* analysis disclosed the damaging function of the p.W486S missense mutation and the high evolutionary conservation of p.W486. These two factors taken together with the confirmed impaired splicing of the c.1338+1G>A mutation demonstrate the possible pathogenic effect of these compound heterozygous *POMT1* mutations in the MDDGC1 of this family.

The *POMT1* gene, mapped to chromosome 9q34.13, spans over 20 kb, contains 20 exons and encodes the protein O‐mannosyltransferase 1 (POMT1) with 747 amino acids. The protein and its homologue POMT2 possess protein O‐mannosyltransferase activity and are putatively involved in O‐mannosyl glycan synthesis, important for muscle, brain and eye development 23, 24, 25. The highly conserved domains of POMT1, protein mannosyltransferase and mannosyl‐IP3R‐RyR (MIR) are involved in the recognition and/or binding of protein substrates, and/or catalysis 26. It is ubiquitously expressed in human embryonic and adult tissues with high expression in foetal brain, pituitary, testis, skeletal muscle and heart 27, 28.

To date, at least 72 mutations in the *POMT1* gene have been described in the literature at the Human Gene Mutation Database (http://www.hgmd.cf.ac.uk/). The disruptive effect of *POMT1* gene mutations can be mediated through the reduced or absent O‐mannosylation of target proteins in a loss‐of‐function mechanism 24. At least 11 *POMT1* gene mutations are identified in MDDGC1 cases, varying from missense and nonsense mutations, through small indel, to gross deletion 1, 23.

The splice‐site mutation c.1338+1G>A in the *POMT1* gene, which is adjacent to the conserved MIR domain, leads to exon 13 being skipped and a reading frame shift and results in a truncated protein. The mutation was previously reported to be responsible for severe type II lissencephaly, classified to MDDGA1, together with a nonsense mutation c.1858C>T (p.R620X) 29. The allelic missense mutation c.1457G>C (p.W486S) in exon 15 coding for the conserved MIR domain, recorded in the dbSNP (rs746849558) with a low frequency, may reduce disease severity, similar to a previous report 9.

This is the first known report of *POMT1* c.1338+1G>A (p.H415Kfs*3) and c.1457G>C (p.W486S) mutations in compound heterozygotes, responsible for the autosomal recessive MDDGC1 in this pedigree. The exon 13‐skipping effect of a splice‐site mutation was first confirmed by RNA analysis. These findings may enlarge phenotype and mutation spectrum of the *POMT1* gene, allowing for greater diagnostic accuracy in exome sequencing and genetic counselling for undiagnosed or ambiguous disorders 30. Phenotypic categorization, accompanied by molecular screening, should facilitate accurate diagnosis, prognosis and genetic counselling.

## Conflict of interest

The authors confirm that there are no conflicts of interest.
